# Surgical checklists: the human factor

**DOI:** 10.1186/1754-9493-7-14

**Published:** 2013-05-14

**Authors:** Paul O’Connor, Catriona Reddin, Michael O’Sullivan, Fergal O’Duffy, Ivan Keogh

**Affiliations:** 1 Department of General Practice, National University of Ireland, Galway, Ireland; 2 School of Medicine National University of Ireland, Galway, Ireland; 3 Department of Surgery National University of Ireland, Galway, Ireland; 4 Academic Department of Otolaryngology, National University of Ireland, Galway, Ireland

**Keywords:** Surgical checklist, Surgery, Patient safety

## Abstract

**Background:**

Surgical checklists has been shown to improve patient safety and teamwork in the operating theatre. However, despite the known benefits of the use of checklists in surgery, in some cases the practical implementation has been found to be less than universal. A questionnaire methodology was used to quantitatively evaluate the attitudes of theatre staff towards a modified version of the World Health Organisation (WHO) surgical checklist with relation to: beliefs about levels of compliance and support, impact on patient safety and teamwork, and barriers to the use of the checklist.

**Methods:**

Using the theory of planned behaviour as a framework, 14 semi-structured interviews were conducted with theatre personnel regarding their attitudes towards, and levels of compliance with, a checklist. Based upon the interviews, a 27-item questionnaire was developed and distribute to all theatre personnel in an Irish hospital.

**Results:**

Responses were obtained from 107 theatre staff (42.6% response rate). Particularly for nurses, the overall attitudes towards the effect of the checklist on safety and teamworking were positive. However, there was a lack of rigour with which the checklist was being applied. Nurses were significantly more sensitive to the barriers to the use of the checklist than anaesthetists or surgeons. Moreover, anaesthetists were not as positively disposed to the surgical checklist as surgeons and nurse. This finding was attributed to the tendency for the checklist to be completed during a period of high workload for the anaesthetists, resulting in a lack of engagement with the process.

**Conclusion:**

In order to improve the rigour with which the surgical checklist is applied, there is a need for: the involvement of all members of the theatre team in the checklist process, demonstrated support for the checklist from senior personnel, on-going education and training, and barriers to the implementation of the checklist to be addressed.

## Introduction

While patient safety problems can occur in non-acute domains of healthcare there are particular risks associated with surgery [1]. It has been found that at least half of all surgical complications are avoidable [2]. Between 1990 and 2010 in the United States there were more than 9,000 surgical ‘never events’ (e.g. retained foreign bodies, wrong-site, wrong-patient, and wrong-procedure surgery). Mortality occurred in 6.6% of patients, permanent injury in 32.9%, and temporary injury in 59.2%. Malpractice payments for these events was $1.3 billion [3]. Recognising the risk to surgical patients, the World Health Organisation (WHO) published guidelines to improve patient safety in the operating theatre [4]. Based upon these guidelines a 19-item checklist was designed with the goal of reducing the rate of major surgical complications [5].

Evaluations of the effects of the WHO surgical checklist have been overwhelmingly positive. The checklist has been shown to reduce mortality in major surgery by 47%, and reduce morbidity by 36% [5]. In a study focused on emergency surgery, there was a 62% relative reduction in mortality, and 36% relative reduction in the complication rate [6]. The WHO checklist has also been found to positively influence the safety culture of theatre personnel [7,8]. However, despite the benefits of the WHO checklist for patient safety, in some cases the practical implementation of the checklist has been found to be less than universal [9], and to decay over time [10].

The purpose of the research reported in this article is to examine attitudes towards an adaptation of the WHO surgical checklist as it has been implemented in an Irish hospital. Although audits at the hospital demonstrated that the checklist is generally being applied, anecdotal evidence suggested that levels of compliance, and attitudes of staff towards, the checklist were mixed.

A questionnaire methodology was used to quantitatively evaluate the attitudes of theatre towards the surgical checklist with relation to: beliefs about levels of compliance and support, impact on patient safety and teamwork, and barriers to the use of the checklist.

### Local implementation of the checklist

A committee was convened by the hospital to introduce a surgical checklist in 2008. The checklist was designed by the committee and then piloted prior to being implemented across all theatres in the hospital in July 2009. There was not any formal staff training in the use of the checklist. However, presentations on the checklist were made at surgical, anaesthetic and nursing forums.

Additions and modifications to the WHO surgical checklist to fit local practices are encouraged [4]. The local ‘pre-operative marking verification checklist’ consists of the following checks, all of which are recorded on a single page that is filed in the patient’s chart.

• Check 1: check patient’s identity, check site marked, and check correct patient labels. Check 1 is then signed by a member of the ward staff, and a member of the reception staff.

• Check 2: check patient’s identity, check documentation to ascertain site, operation surgeon/senior member of team check, and marks site if not completed on ward. Check 2 is then signed by the consultant surgeon or senior member of the team.

• Check 3: time out procedure is conducted to confirm the correct patient, marking of the correct site, agreement on procedure to be performed, correct patient position, and the availability of correct implants/imaging studies/histology (as appropriate). Check 3 is signed by the consultant surgeon/senior team member, senior anaesthetist, and circulating nurse.

Checks 2 and 3 are generally carried out in the operating theatre once the patient has been anaesthetised, and are the main focus of this study.

## Method

### Questionnaire development

Following approval by the Ethics Board Chairman of Galway University Hospitals for the study, semi-structured interviews were carried out with 14 members of theatre staff: six surgeons (three Consultants, two Senior House Officers (SHOs), and one Registrar), four anaesthetists, (two Consultants, and two Registrars) and four theatre nurses. The interview protocol was based upon the theory of planned behaviour [11]. The theory of planned behaviour proposes that an individual’s behaviour is driven by behavioural intentions. Behavioural intentions are a function of an individual's attitude toward the behaviour (e.g. do they think the use of the checklist is worthwhile?), the subjective norms surrounding the performance of the behaviour (e.g. is the checklist widely used at the hospital?), and the individual's perception of the ease with which the behaviour can be performed (e.g. are you able to initiate the use of the checklist prior to beginning an operation?).

Based upon the 14 semi-structured interviews, a 27-item questionnaire was developed. The questionnaire consisted of five subscales: attitudes towards hospital norms on the use of the checklist (five items), the impact of the checklist on safety and teamwork (five items), support of the checklist from specific groups (six items), intent to initiate the checklist (2 items), and barriers to the use of the checklist (five items). Also included were: an item asking whether there was a difference between the surgical checklist used at the hospital and the WHO surgical checklist, an open ended question allowing the respondent to identify additional barriers to the use of the checklist, and two demographic questions. All responses to the attitude items were on a five-point scale from 1 (strongly disagree), to 5 (strongly agree). The respondents were also given the option of selecting ‘don't know’. The questionnaire is available for download from http://www.midss.ie.

### Procedure

Paper copies of the questionnaire were distributed to theatre staff in the theatre coffee room. These questionnaires were distributed, and collected by a member of the research team. In addition, a link to a web based copy of the questionnaire was also circulated via email to all theatre staff. A reminder email was sent four weeks after the initial email request. The responses were anonymous with no identifiable information collected.

### Data analysis

The individual responses to the Likert scale questionnaire items were used to calculate the sub-scale scores. The five sub-scale scores were derived by calculating the mean response of the item that made up each sub-scale. Cronbach’s alpha was then used to assess the internal consistency of each of the five sub-scales. An alpha score of 0.7 or greater is regarded as indicative of an acceptable level of internal consistency [12], but low thresholds are sometimes quoted in the literature.

Two statistical tests were carried out in the analysis of the data using the sub-scale scores. A Kruskal-Wallis test (the nonparametric version of the Analysis of Variance) [13] was conducted to evaluate differences among the three roles (surgeon, anaesthetist, and nurse) on median differences in responses for each of the factors. In this test a chi-square (χ^2^) statistic is used to evaluate differences in mean ranks to assess the null hypothesis that the medians are equal across the groups. For surgeons and anaesthetists only, two-way ANOVAs were carried out based upon role (surgeon or anaesthetist), and seniority (consultant versus non-consultant).

For the open-ended question concerned with barriers to the use of the checklist, two raters independently categorised the comments, with the inter-rater reliability measured using Cohen’s Kappa. A Kappa of 0.8 or higher is considered to be indicative of a perfect agreement between the raters [14].

### Participants

Responses were obtained from 41 surgeons (40.6% response rate), 33 anaesthetists (58.9% response rate), and 33 theatre nurses (34.7% response rate). Of the medical staff, 23 were Consultants, 50 were either SHOs or Registrars, with data on role missing from one respondent. Of the nurses, three responses were obtained from Clinical Nurse Managers, with the remaining responses from staff nurses.

## Results

Table 1 summarises the responses of the participants to each of the items in the questionnaire separated by role in theatre and shows the mode and inter quartile range (IQR; a measure of the spread of the data). The sub-scale scores, and the Cronbach’s alpha value of each sub-scale, are shown in Table 2.

**Table 1:** Summary of attitudinal responses (responses range from 1 ‘strongly disagree’ to 5 (strongly agree’)

**Sub-scale**	**Attitudinal items**	**Surgeon (n=41)**		**Anaesthetist (n= 33)**		**Nurse (n=33)**	
		**Mode**	**IQR**	**Mode**	**IQR**	**Mode**	**IQR**
	There is little difference between the surgical checklist at GUH and the WHO surgical checklist.	3	2	1	3.25	2	2.75
**Norms**	The complete checklist is used for every procedure in every theatre at UHG.	2	3	1	3	5	1
	The complete checklist is used for every procedure in which I am involved in theatre.	5	1.5	1	3	5	1
	When the checklist is being carried out, everyone in theatre stops what they are doing and listens until it is completed.	1	3	1	1	1	1.5
	Sometimes sections of the checklist are not completed.*	4	2	4	2	4	1
	The individual who signs the checklist personally ensures that the relevant steps have been completed.	5	1.5	4	2	4	1
**Impact on teamwork & safety**	I believe that failing to use the checklist is poor professional practice.	5	1	5	1	5	0
	I believe using the checklist reduces the likelihood of human error.	5	0.75	5	1	5	0
	I believe using the checklist improves patient safety.	5	0.5	5	1	5	0
	I believe using the checklist improves teamwork in theatre.	5	1	5	1	5	1
	The use of the checklist should be mandatory for every case.	5	1	5	1	5	0
**Support**	Surgical personnel support the use of the checklist.	5	1	4	1	5	5
	Anaesthetic personnel support the use of the checklist.	4	2	4	1	5	4
	Nursing staff support the use of the checklist.	5	0	4	1	5	5
	Senior theatre personnel support the use of the checklist.	5	2	4	1	5	5
	Junior theatre personnel support the use of the checklist.	5	1	4	1	5	5
	Management support the use of the checklist.	5	2	5	1	5	5
**Initiate**	I have initiated the use of the checklist in the past.	5	2	1	3	5	0
	I intend to initiate the use of the checklist in the future.	5	1	3	1.75	5	0
**Barriers**	The requirement for signatures.	4	2.5	5	3	5	1
	Lack of assertiveness of staff.	4	2	4	2.5	4	1
	Lack of time.	4	2	4	2.75	4	1
	Lack of training.	4	2	4	2	4	1.5
	The lack of an electronic version of the checklist.	3	2	5	3	3	2

*reverse worded item.

**Table 2:** Summary data for the sub-scales

**Sub-scales**	**Alpha**	**Surgeon**		**Anaesthetist**		**Nurse**	
		**Mean**	**St dev**	**Mean**	**St dev**	**Mean**	**St dev**
Norms	0.70	3.36	0.88	2.58	0.95	3.35	0.67
Impact on teamwork & safety	0.84	4.46	0.72	4.43	0.71	4.86	0.20
Support	0.73	4.23	0.62	3.94	0.63	4.36	0.62
Initiate	0.87	4.10	1.13	3.28	1.26	4.71	0.59
Barriers	0.56	3.03	0.80	3.34	0.84	3.85	0.67

### Between group comparisons

The Kruskal-Wallis test, which corrected for tied ranks, was significant for all of the sub-scales: ‘norms’, χ^2^ (2, N= 107)= 13.95, p<.05); ‘impact on teamwork and safety’, χ^2^ (2, N= 107)= 7.50, p<.05); ‘support’, (χ^2^ (2, N= 107)= 8.34, p<.05); ‘initiate’, χ^2^ (2, N= 105)= 24.67, p<.05); and ‘barriers’, χ^2^ (2, N= 107)= 20.54, p<.05). Follow-up tests were conducted to evaluate pairwise differences among the three groups for each sub-scale, controlling for Type I error across tests by using the Bonferroni approach.

For the ‘norms’ sub-scale anaesthetists had significantly more negative attitudes than surgeons and nurses. For the ‘impact on teamwork and safety’ and ‘support’ sub-scales anaesthetists had significantly more negative attitudes than nurses. For the ‘initiate’ sub-scale, anaesthetists scored significantly lower than nurses and surgeons. Also, surgeons scored significantly lower than nurses on the ‘initiate’ sub-scale. For the ‘barriers’ sub-scale, nurses were significantly more sensitive to the barriers to the use of the checklist than surgeons and anesthetists.

For surgeons and anaesthetists only, two-way ANOVAs were carried out based upon role (surgeon or anaesthetist), and seniority (consultant versus non-consultant). It was necessary to group Registrars and SHOs together due to the small sample size. There were significant main effects of role for ‘norms’ (F(1, 74)= 14.40, p<.05), ‘support’ (F(1, 74)= 4.23, p<.05), and ‘initiate’ (F(1, 74)= 8.50, p<.05). For ‘intent’ there was a significant main effect of seniority (F(1, 74)= 4.72, p<.05). None of the interactions between the variables were significant. Unsurprisingly, consultants were significantly more likely to initiate the use of the checklist than more junior doctors.

### Open-ended statements

Participants were given the opportunity to identify addition barriers to the use of the checklist that were not addressed in the questionnaire. Two raters independently coded the 42 comments into one of seven categories (see Table 3). The percentage agreement was 90% between the two rater (Cohen’s kappa= 0.88). The four statements in which the raters did not agree were discussed, and an agreement reached on the appropriate category.

**Table 3:** Categorisation, and examples, of open-ended questions regarding additional barriers to the use of the checklist

**Category**	**Total number of comments assigned to category**	**Example**
Method of implementation	13	• Too much paper and signatures. Lack of use of x-rays and pathology.
Lack of teamwork	9	• Need for complete staff involvement. Staff frequently do not pay attention or demand silence and attention of all staff.
Timing	6	• Checklist frequently carried out when anaesthetists are busy with airway etc.
Lack of education	5	• Lack of education regarding benefits.
Lack of senior support	4	• Intimidation by senior staff.
Lack of redundancy	3	• Lack of repeating checklist after patient is positioned.
Too much redundancy	2	• Too much repetition.

## Discussion

The purpose of this study was to use a questionnaire methodology to obtain information on attitudes to a surgical checklist as administered in an Irish hospital. There would appear to be considerable variability in the implementation of the checklist and evidence that there may be a lack of rigour in its application.

Although it is positive finding that the checklist is being used, a lack of rigour in it’s application could lead to a false sense of security, and actually compromise safety and teamworking [15]. A particular area worth highlighting is the low levels of compliance with the ‘time out’ prior to surgery. The lack of adherence to the ‘time out’ has been identified as an issue in other studies. In a survey of Irish surgeons it was found that only 7.3% of surgeons stated that an adequate pre-operative team brief was frequently conducted [16].

Although this might not be expected from the reported levels of compliance, the overall attitudes towards the checklist from the respondents were overwhelmingly positive. This finding is consistent with other studies examining attitudes towards a surgical checklist [5,7]. Nurses are the most supportive members of the theatre team for the use of the checklist. Other studies also imply that nurses are particularly positively disposed to the checklist as compared to other theatre personnel [17].

### Barriers to the use of the checklist

Likely due to the fact the nurses have the responsibility for completing the checklist; they are significantly more sensitive to the barriers to completing the checklist than surgeons and anaesthetist. In particular, nurses believed that the requirement for signatures, lack of time, and assertiveness of staff were barriers to the completion of the checklist to a greater extent than surgeons or anaesthetists. Previous research in the operating theatre has found that nurses are more sensitive to issues of poor teamworking that surgeons or anaesthetists [18]. It has also been reported elsewhere that the steep hierarchy of the surgical team serves as a barrier to nurses being checklist coordinators, despite the fact that nurses have taken on the responsibility of ensuring consistent use of the checklist [17]. Commonly mentioned additional barriers provided by participants in the current study included: issues with the implementation of the checklist itself, poor teamwork, and the timing of when the checklist is carried out.

The timing of when the checklist is completed would appear to be a particular problem for anaesthetists. The checklist tends to be completed during a periods of high workload for the anaesthetist prior to surgery. Therefore, they are not able to focus on what is happening with the checklist. All of the open-ended responses on barriers concerned with the poor timing of the completion of the checklist were made by anaesthetists. Other research has also found timing to be an issue [17], and that checklists can create inter-professional tensions as a result of factors such as when the checklist is completed [19].

### Recommendations for improving checklist compliance

Although the recommendation for improving checklist compliance are based upon the findings from the current study, they draw upon the broader literature discussing the implementation of surgical checklists. Therefore, these recommendations have relevance to other hospitals in which the WHO surgical checklist, or a local adaptation, have been implemented.

#### Need to involve all members of the theatre team in the checklist process

All members of the theatre team must be involved in the checklist procedure such that it is a true multidisciplinary intervention. Although anaesthetists have been acknowledged as the leading medical specialty in addressing issues of patient safety [20], in the current study their attitudes towards the application of the checklist were less positive than those of surgeons or nurses. It is recommended that discussions are held with anaesthetists in order to identify a time in which they are able to be involved in the checklist process. It is suggested that the checklist should be completed prior to the anaesthetising of the patient. In some cases this would have the added safety benefit of allowing the patient to be part of the checklist process.

#### Demonstrated support for checklist adherence from senior personnel

Giving the job of initiating the checklist to the circulating nurse reduces the likelihood of diffusion of responsibility whereby a person is less likely to take responsibility for action or inaction when others are present. However, the circulating nurse must be supported in this role by other members of the theatre team- particularly those in more senior positions. There is also a need to support the period of ‘time out’ to allow the checklist to be completed, and ensure that all members of the theatre team are engaged in the process.

In the current study, although the majority of the respondents believed that management support the use of the checklist, 10% of the open-ended comments identified a lack of senior support as a barrier to the use of the checklist. It is suggested that clinicians in a Medical Director position need to set the tone for this senior support through ‘safety leadership walkarounds’ in which they help champion the consistent use of the checklist [15]. For example, visible leadership has been found to be the most important factor in implementing teamwork training techniques and principles in a healthcare setting [21].

#### On-going education and training

Training has been found to raise the frequency in the implementation of surgical checklists from 8% to 97% [22]. However, there is a need for continued reinforcement of safety initiatives such as the implementation of the checklist. A one-off training programme will have limited effectiveness. In a study carried out in a French hospital the frequency in the implementation of the checklist dropped from 88% to 76% in the first year of use [10]. It is recognised that opportunities for training are limited, and not something that the respondents to the questionnaire in the current study generally felt was required. However, it is recommended that each department has a designated ‘checklist champion’ who is responsible for providing training and reinforcing the use of the checklist in theatre.

#### Address the barriers to the implementation of the checklist

Finally, it is suggested that there is also a need to re-examine the checklist itself. In the version of the checklist used in this study there was a requirement to obtain signatures from the surgeon, anaesthetist, and circulating nurse. In particular, the nurses recognised this as a barrier to the completion of the checklist. It could be argued that if the circulating nurse is responsible for the task, only his/her signature should be necessary, with the surgeon and anaesthetist verbally confirming it has been completed. Perhaps the requirement for signatures could be re-examined, and used as a ‘carrot’ to encourage more rigorous use of the checklist. To illustrate, pilot a version of the checklist without the requirement for all three signatures, if compliance improves with this adapted version of the checklist then it will be adopted permanently.

### Study limitations

There are two main limitations to the research reported in this paper. Firstly, as the paper reports attitudes of theatre staff from one hospital to a specific locally adapted version of the WHO surgical checklist it could be argued that the findings are not generalizable. Although the checklist examined in the current study differs from the actual WHO surgical checklist, the overall goals of ensuring that it is the correct patient, for the correct surgery, on the correct site are common to both. Moreover, regardless of the exact steps in the checklist issues of timing, senior support, assertiveness, acceptance, and adherence are common issues identified with surgical checklists [9,10,17,23], as well as the use of checklists in other healthcare domains [24].

The second limitation is the response rate. Although the response rate is not atypical of questionnaire studies of this type, [7,16,25] the findings should be regarded with some degree of caution. Nevertheless, the data were not inconsistent with other studies of attitudes towards surgical checklists [7,9,10,17,19], and the study was broadly representative of the personnel that work in theatre.

## Conclusions

The surgical checklist is potentially a very effective system for avoiding many potential ‘never events’ in surgery. However, a recognition by theatre staff of the benefits of the checklist for patient safety and teamworking, does not necessarily lead to rigorous application. There is a need to consider, and address, sociocultural issues such as the workload of particular team members, timing of when the checklist is carried out, perceived support from senior personnel, and the empowerment of staff to complete the checklist.

## Competing interests

The authors declare that they have no competing interests.

## Authors’ contributions

POC participated in the design of the study, data analysis, and preparation and editing of the manuscript. CR, MOS, and FOD carried out the data collection, provided input to the interpretation of the results, and reviewed the various drafts of the manuscript. IK participated in the design of the study, interpretation of the results, and reviewed the various drafts of the manuscript. All authors read and approved the final manuscript.
